# Supplementary material for: Reliability and validity of an equanimity questionnaire: the two-factor equanimity scale (EQUA-S)
Source: PeerJ. 2020 Jul 7;8:e9405. doi: 10.7717/peerj.9405 (PMC7350915; doi:10.7717/peerj.9405)
Supplement: Supplemental Information 1 [file peerj-08-9405-s001.pdf]

Partie B : Evaluez chacune des propositions ci-dessous en utilisant l'échelle proposée. Cochez le chiffre qui correspond le mieux à ce qui est généralement vrai pour vous. Ne donnez qu'une réponse par proposition.

Jamais ou très rarement vrai 1	Rarement vrai 2	Parfois vrai 3	Souvent vrai 4	Très souvent ou toujours vrai 5

1. Lorsque j'anticipe de faire quelque chose de plaisant, je ne pense qu'à ça.	1	2	3	4	5
2. Lorsque j'anticipe quelque chose ou une situation que j'aime, je suis très excité(e).	1	2	3	4	5
3. Je suis très déçu(e) lorsqu'un évènement que j'attendais tombe à l'eau.	1	2	3	4	5
4. Lorsque je suis attiré(e) par un objet qui me fait envie, je ressens une forte attraction pour l'obtenir rapidement	1	2	3	4	5
5. Je suis facilement contrarié(e) par un désagrément	1	2	3	4	5
6. Cela me met facilement de mauvaise humeur si je suis contraint de faire une tâche que je trouve fastidieuse (comme faire la vaisselle ou récurer les toilettes)	1	2	3	4	5
7. Je suis très excité(e) lorsqu'il m'arrive ou que l'on me donne quelque chose de plaisant (comme une bonne surprise ou un cadeau)	1	2	3	4	5
8. Je souhaite souvent prolonger les moments où je ressens un fort plaisir	1	2	3	4	5
9. J'ai du mal à m'arrêter lorsque je fais quelque chose que j'aime	1	2	3	4	5
10. Je ne me laisse pas facilement submerger/envahir par mes émotions	1	2	3	4	5
11. Quoi qu'il arrive, je reste serein(e)	1	2	3	4	5
12. Je ne suis pas facilement perturbé(e) par un imprévu	1	2	3	4	5
13. Je réagis en suivant mes désirs/pulsions	1	2	3	4	5
14. J'ai du mal à tolérer les émotions inconfortables	1	2	3	4	5
15. J'ai du mal à réfréner/maitriser certaines de mes pulsions	1	2	3	4	5
16. Je peux facilement me laisser emporter par une contrariété	1	2	3	4	5
17. Si quelque chose m'agace, je suis incapable de ne pas réagir	1	2	3	4	5
18. Je ne suis pas dépendant(e) de mes envies	1	2	3	4	5
19. Je ressens que je suis une personne calme, même dans des moments de stress et tension	1	2	3	4	5
20. Les situations de stress me perturbent émotionnellement.	1	2	3	4	5
21. Il est difficile pour moi d'être serein(e) pendant les moments difficiles de la vie quotidienne.	1	2	3	4	5
22. Je ressens que les problèmes dans ma vie sont temporaires et qu'il existe des solutions.	1	2	3	4	5
23. Je n'ai pas assez de patience et de tolérance avec moi-même et les autres.	1	2	3	4	5

Jamais ou très rarement vrai 1	Rarement vrai 2	Parfois vrai 3	Souvent vrai 4	Très souvent ou toujours vrai 5

24. Je me considère comme quelqu'un de stable émotionnellement et très calme.	1	2	3	4	5
25. Je ne peux pas laisser un problème bénin sans essayer d'y apporter une solution immédiate	1	2	3	4	5
26. Je suis très déstabilisé(e) lorsqu'on me critique	1	2	3	4	5
27. Cela m'énerve lorsqu'une personne est ingrate envers moi	1	2	3	4	5
28. Cela me fait toujours plaisir de recevoir des louanges	1	2	3	4	5
29. Peu importe que je gagne ou perde à un jeu, pour moi cela n'a pas beaucoup d'importance	1	2	3	4	5
30. Cela me rend très fier(e) lorsque les autres me flattent	1	2	3	4	5
31. Je suis très sensible à la critique	1	2	3	4	5
32. Après ma mort, il est important pour moi que l'on se souvienne de moi et que je ne tombe pas dans l'oubli	1	2	3	4	5
33. Lorsqu'on me grille la priorité en voiture ou comme piéton, cela m'agace au point d'être capable de dire des gros mots ou des insultes	1	2	3	4	5
34. Lorsque je vois ou vis une injustice, je perds tous mes moyens	1	2	3	4	5
35. Je fais tout pour éviter les choses ou les situations que je n'aime pas.	1	2	3	4	5
36. Lorsque je suis obligé(e) de faire des tâches administratives qui ne me plaisent pas, je reste calme	1	2	3	4	5
37. Si je dois attendre longtemps dans une file, je reste serein(e)	1	2	3	4	5
38. J'essaie autant que possible d'obtenir les choses ou les situations que je convoite	1	2	3	4	5
39. Lorsque j'apprends que je ne pourrais pas faire ce dont j'avais envie, je ressens des émotions qui me perturbent	1	2	3	4	5
40. Si je m'isolais dans un coin silencieux pour être tranquille, je serais perturbé(e) si quelqu'un venait faire du bruit et rompre le silence.	1	2	3	4	5
41. Cela peut finir par m'énerver lorsque quelqu'un ne comprend pas ce que je veux dire.	1	2	3	4	5
42. Cela ne me contrarie pas vraiment lorsqu'une personne me dit qu'elle n'aime pas ce que j'ai fait/accompli.	1	2	3	4	5

1. When I anticipate doing something fun, that's all I think about.
2. When I anticipate something or a situation that I like, I get very excited.
3. I am very disappointed when something I was expecting falls through.
4. When I am attracted to something I want, I feel a strong attraction to get it quickly.
5. I am easily annoyed by an inconvenience
6. It easily puts me in a bad mood if I am forced to do a task that I find tedious (such as washing dishes or scrubbing toilets).
7. I am very excited when something pleasant happens to me or is given to me (such as a nice surprise or gift).
8. I often wish to prolong the moments where I feel a strong pleasure
9. I find it hard to stop when I'm doing something I love.
10. I don't easily get overwhelmed/invaded by my emotions.
11. Whatever happens, I remain serene
12. I am not easily disturbed by an unforeseen event.
13. I react by following my desires/pulses
14. I find it hard to tolerate uncomfortable emotions
15. I have difficulty restraining/controlling some of my impulses.
16. I can easily get carried away by a vexation
17. If something annoys me, I am incapable of not reacting.
18. I am not addicted to my desires
19. I feel that I am a calm person, even in times of stress and tension.
20. Stress situations upset me emotionally.
21. It is difficult for me to be serene during the difficult moments of everyday life.
22. I feel that the problems in my life are temporary and that there are solutions.
23. I do not have enough patience and tolerance with myself and others.
24. I consider myself to be emotionally stable and very calm.
25. I can't let a benign problem go unresolved without trying to find an immediate solution.
26. I am very unsettled when I am criticized.
27. It annoys me when someone is ungrateful to me.
28. It always pleases me to receive praise
29. It doesn't matter if I win or lose in a game, it doesn't really matter to me.
30. It makes me very proud when others flatter me.
31. I am very sensitive to criticism
32. After my death, it is important to me that I am remembered and not forgotten.
33. When I am given the right of way in a car or as a pedestrian, it annoys me to the point that I am able to say bad words or insults.
34. When I see or experience an injustice, I lose all my means.
35. I do everything I can to avoid things or situations I don't like.
36. When I am forced to do administrative tasks that I don't like, I stay calm.
37. If I have to wait a long time in a line, I stay calm.
38. I try as much as I can to get the things or situations I covet
39. When I learn that I won't be able to do what I wanted to do, I feel emotions that disturb me
40. If I isolated myself in a quiet corner to be quiet, I would be disturbed if someone came to make noise and break the silence.
41. It can end up annoying me when someone does not understand what I mean.
42. It doesn't really upset me when someone tells me that they don't like what I've done/fulfilled.
